# Commentary on: Labels, cognomes, and cyclic computation: an ethological perspective

**DOI:** 10.3389/fpsyg.2015.00784

**Published:** 2015-06-11

**Authors:** Cedric Boeckx, Constantina Theofanopoulou

**Affiliations:** ^1^Department of General Linguistics, Universitat de BarcelonaBarcelona, Spain; ^2^Catalan Institute for Research and Advanced StudiesBarcelona, Spain

**Keywords:** labeling, cognome, language evolution, computation, animal cognition, cognitive phylogenies

Murphy (2015) seeks to gain insight into the phylogeny of the human language faculty by adopting a computational perspective. After surveying the ethology literature in an attempt to isolate what is lacking in other species at the computational level, Murphy claims that the process of Labeling, well studied in theoretical linguistics, is what is specific to human language.

We applaud Murphy's effort to address the issue of language phylogeny in such a computationally explicit fashion, but we would like to take this opportunity to highlight a few challenges to building adequate cognitive phylogenies that Murphy does not seem to appreciate enough.

While Murphy's critical remarks toward the “Chomsky hierarchy” as a tool to build biologically-sound cognitive phylogenies (Fitch, 2015) echoes some of our own concerns (Boeckx, 2013; Benítez-Burraco and Boeckx, 2014), we find his appeal to computational principles like Labeling to be equally inadequate. Quite apart from our skepticism coming from narrowly linguistic considerations surrounding Labeling (Boeckx, 2014), our main reason for not siding with Murphy is that issues of cognitive phylogenies must be firmly grounded in comparative studies. But it is well-known that linguistic principles of the sort Murphy trades in resist meaningful comparison across cognitive domains and species.

As Newport (2010: p. 282) has correctly observed, “the generative tradition in language has given us an elegant and detailed articulation of how these principles work themselves out in language; whether the same principles apply in detail to any other domain remains to be seen, since few comparably sophisticated analyses have ever been done of other complex cognitive domains.” The generative tradition took some 50 years to arrive at the sophisticated level of computational characterization that Murphy seeks to exploit. Nothing like it exists in other domains of human cognition, let alone in other species. Accordingly, how are we to determine if, say, the “cognome” of baboons contains the Labeling operation when they conceptualize dominance hierarchies? It is not that questions of this type are meaningless, it is just that they are inapplicable in practice, casting doubt on the current feasibility of what Murphy calls computational ethology.

We also find it difficult to believe that operations like Labeling are formulated at the right level of granularity to enable the formulation of linking hypotheses between the cognome and the dynome, connectome, and genome. As argued in Boeckx and Theofanopoulou (2014), notions of computation grounded in brain processes, of the sort advocated by Buzsáki (2010) and Buzsáki and Watson (2012), stand a much better chance of providing the right bridging tools between mind and brain. They also can rely on the conservation of brain rhythms across a wide range of species (Buzsáki et al., 2013) to begin to draw meaningful comparisons across species and cognitive domains.

Despite some supporting remarks toward our work, we think that Murphy fails to truly appreciate both the necessity and the primacy of cross-disciplinary, multi-dimensional hypotheses of the sort we have advocated (Boeckx and Theofanopoulou, 2014). In the absence of these, it is quite natural to weaken the Darwinian notion of continuity (as Murphy in fact does with his Weak Continuity Hypothesis), because principles at one level (say, the phenome or cognome) tend to be so specific as to render descent scenarios difficult to articulate. And it is not only this difficulty that worries us, but mainly the fact that phenotypic diversity across species reveals only an apparent gulf (hence, apparent discontinuity) without discernible effects on the cognome. Attempts to detect how such effects percolate downstream succeed to the extent that some traits can indeed be decomposed with a “top-down” approach. But, in our view, it is clear that what is needed instead is a “bottom-up” approach (De Waal and Ferrari, 2010), where sharp differences in the phenome would be conceived as confluences of a hodgepodge of recognizable, reconstructed features. From this standpoint, behavioral-phenotypic experiments across species would only serve as “bootstrapping” bottom-up hypotheses. We are thus convinced that evolution's “tinkering” character—in the spirit of Darwin's notion of “descent”—is to be found in those deeper, more elementary, widely shared traits, as opposed to apparent-species-specific behaviors. It is in fact this specificity that tends to promote cladistic formulations of cognitive phylogenies (Fitch et al., 2010). But once multi-dimensional hypotheses are formulated, such traditional phylogenetic representations become untenable (Theofanopoulou, 2015; Theofanopoulou and Boeckx, in preparation).

Consider, for example, the origin of “syllables” in human language, an issue very much related to Labeling. Syllables in language are distinct from “syllables” in the vocal outputs of other species (Samuels, 2011). Accordingly, we could be tempted to posit a human-specific operation—Syllabify—to capture this fact. But work by Ghazanfar and Takahashi (2014) has revealed that a decomposition of syllables in terms of brain rhythms (dynome) favors an evolutionary scenario according to which the mechanism of syllabification is rooted in the mechanism of lip-smacking attested in non-linguistic primates. Computationally speaking, lip-smacking and speech are distinct at the phenome level, but mechanistically (in the dynome) they converge, and provide the basis for elementary cognitive functions (attention, working memory; see Martins and Boeckx, 2014). Murphy's computational ethology approach would be unable to capture it, but the neuroethological approach of Ghazanfar and colleagues does, by resorting to elementary and generic operations framed in brain terms.

In sum, we fully endorse the “divide-and-conquer” approach to cognitive traits that Murphy adopts, but we want to stress the need to recognize that such an approach will only be successful if the “divide” step is combined with a linking step across phenome, cognome, dynome, connectome, and genome. In Aristotle's terms, it is this bridging step that will make us grasp both the Continuity—“in essence” and the Discontinuity—“in appearance.”

## Conflict of interest statement

The authors declare that the research was conducted in the absence of any commercial or financial relationships that could be construed as a potential conflict of interest.
